# Association between Vitamin D and Adiponectin and Its Relationship with Body Mass Index: The META-Health Study

**DOI:** 10.3389/fpubh.2014.00193

**Published:** 2014-10-14

**Authors:** Aurelian Bidulescu, Alanna A. Morris, Neli Stoyanova, Yuan-Xiang Meng, Viola Vaccarino, Arshed A. Quyyumi, Gary H. Gibbons

**Affiliations:** ^1^Department of Community Health and Preventive Medicine, Cardiovascular Research Institute, Morehouse School of Medicine, Atlanta, GA, USA; ^2^Department of Medicine, Emory School of Medicine, Atlanta, GA, USA; ^3^Department of Family Medicine, Morehouse School of Medicine, Atlanta, GA, USA; ^4^Department of Epidemiology, Emory University Rollins School of Public Health, Atlanta, GA, USA; ^5^Division of Cardiology, Emory Clinical Cardiovascular Research Institute, Emory School of Medicine, Atlanta, GA, USA; ^6^National Heart, Lung, and Blood Institute, National Institutes of Health, Bethesda, MD, USA

**Keywords:** vitamin D, adiponectin, obesity, minorities, African-Americans, obesity

## Abstract

**Background:** Low vitamin D and adiponectin levels are both associated with obesity and cardiovascular disease. Previous studies have indicated that vitamin D levels are directly associated with adiponectin, and that this association varies across body mass index (BMI) categories; stronger with increasing BMI. Few studies examined this association in African-Americans (AA), known to have lower levels of vitamin D and adiponectin, and in whites.

**Methods:** We assessed whether serum vitamin D is associated with serum adiponectin in a biracial population-based sample. Cross-sectional analyses were performed on 426 non-diabetic participants (218 whites and 208 AA) from the META-Health Study, a random sample from the metro Atlanta. Age-adjusted correlations and multivariable linear regression were used for analyses. We investigated the effect modification of the BMI categories of lean, overweight, and obese as defined by standard cut-points (25 and 30 kg/m^2^).

**Results:** The mean (SD) age of our study sample was 50.5 (9) years. The mean (SD) levels of vitamin D were 27.4 (9.8) ng/mL in white women, 25.5 (9.3) ng/mL in white men, 16.9 (7.3) ng/mL in AA women, and 18.8 (7.3) ng/mL in AA men. The mean (SD) levels of adiponectin were 17.0 (17.1) μg/mL in white women, 9.9 (11.3) μg/mL in white men, 6.6 (4.8) μg/mL in AA women, and 9.4 (11.6) μg/mL in AA men. Among lean white women (*n* = 63), there was a significant direct association between vitamin D and adiponectin (β = 0.02, *p* = 0.04) after adjustment for age, systolic blood pressure, HDL-cholesterol, triglycerides, income, and season of blood drawing. On the contrary, in lean AA women (*n* = 23), there was a significant inverse association (β = −0.06, *p* = 0.01).

**Conclusion:** The association of vitamin D and adiponectin is dependent on race, gender, and BMI category. Among lean white women, there was a significant direct association, whereas in lean AA women the association was inverse. No association was present among obese individuals.

## Introduction

Obesity is a global epidemic that is associated with low levels of vitamin D and adiponectin, cardiovascular disease, and death (1–5). Vitamin D deficiency, as reflected by circulating 25-hydroxyvitamin D levels <20 ng/mL, is prevalent in as many as half of middle-aged to elderly adults in developed countries (6). Adiponectin, the most abundant adipokine, has been suggested to play an important favorable role in atherosclerosis, endothelial inflammation, myocardial remodeling, and several of the cardiometabolic risk factors (7, 8). Low vitamin D and adiponectin levels are both associated with obesity and cardiovascular disease (9–11). Besides the favorable effect of both vitamin D and adiponectin on insulin resistance (12), vitamin D is an essential steroid metabolite with multiple metabolic effects that includes a negative regulation of the adipose-tissue renin–angiotensin system supposed to inhibit adiponectin secretion (13).

Previous studies have indicated that vitamin D levels are directly associated with adiponectin, and that this association varies across body mass index (BMI) categories, stronger with increasing BMI (14–17). Because both low vitamin D and low-adiponectin levels are associated with increased obesity, the association vitamin D – adiponectin might represent an explanation for the increased cardiovascular risk in obesity. Few studies examined this association in African-Americans (AA), known to have lower levels of vitamin D and adiponectin, and in whites (17).

The present study was designed to assess whether serum vitamin D is associated with serum adiponectin in a biracial population-based sample of whites and AA. We aimed also to determine if race and gender modify the association between vitamin D and adiponectin. Because a higher adiponectin level may reduce cardiovascular risk, studies to evaluate the association of vitamin D and circulating adiponectin, especially in obesity, are warranted.

## Materials and Methods

### Study population

The META-Health Study (Morehouse and Emory Team up to eliminate cardiovascular health disparities) is a two-stage study including a random digit dialing (RDD) interview of self-identified white and AA residents of metro Atlanta (Cobb, DeKalb, Fulton, and Gwinnett counties of Georgia), aged 30–66 years, followed by a clinic visit with detailed testing in a subsample (18). The employed sample design was based mainly on the RDD methodology where sample telephone numbers were selected in 100-series telephone banks containing at least one listed household. Nine calls were placed before deciding a party is “unreachable.” Three day attempts, three night attempts, and three weekend attempts were made. The sampling used for this project follows the behavioral risk factor surveillance system (BRFSS) standard for participating area sample designs. Sample records must be justifiable as a probability sample of all households with telephones.

A total of 3,391 individuals were interviewed by phone. All individuals interviewed were asked to come in for a clinic visit to either the Emory or Morehouse Schools of Medicine. Of these, 219 white and 240 AA men and women were examined at the subsequent clinic visit from December 2005 to October 2009 and had adiponectin and vitamin levels measured, constituting approximately 14% of those interviewed by phone. Pregnant women and subjects with acute illnesses were excluded.

We further excluded individuals with missing or incomplete data in any study variables (*n* = 33). Therefore, 218 white (71 men and 135 women) and 208 AA (71 men and 137 women) persons were included in the analysis. Compared to those who were included in the analysis, there was a lower number of AA (43 vs. 49%), but similar percentage of women (66 vs. 64%) in the 3,391 participants that were initially phone-interviewed. This study was approved by the Emory and Morehouse institutional review boards and all participants gave informed consent.

### Covariates

Age, race, and gender were self-identified. Participants were instructed to fast and to refrain from smoking for 12 h before the study visit. During the clinic visit, height was measured with a Portable Shorr Height Measuring Board. All jewelry and hair dressings were removed and participants were left wearing a disposable lightweight gown and shoes, both provided by the study. Participants were asked to stand straight with their back against the measuring board, Frank line horizontal, and heels close together and legs straight. Height was recorded in centimeters to 0.1 cm. Body weight was measured with the S 6600 High-Capacity Floor Scale and weight was recorded in kilograms rounding to the nearest 0.1 kg. From these data we computed the BMI [BMI = weight (kg)/height(m)^2^). BMI was categorized utilizing a standard classification of normal weight (BMI ≤24.9 kg/m^2^), overweight (25.0 ≤ BMI <30), and obesity (BMI ≥30 kg/m^2^). Blood pressure was determined as the average of three measurements taken after 5 min of silent resting. Lipid variables [high-density lipoprotein cholesterol (HDL-C) and triglycerides (TG)], fasting plasma glucose (FPG), and fasting insulin (FI) were measured using standard laboratory techniques. Insulin resistance status was estimated with the homeostasis model assessment (HOMA-IR), as (FPG × FI)/22.5 (19).

### Vitamin D and adiponectin measurements

Venous blood samples were withdrawn from each subject at baseline examination after more than 8 h of fasting as described elsewhere (18). Serum vitamin D (25-hydroxy-vitamin D) was measured by a liquid chromatography, tandem mass spectrometry (LC/MS/MS) procedure (20). The extraction was done via protein precipitation, and the separation via high-performance liquid chromatography (HPLC). Adiponectin concentration was measured as total adiponectin by an ELISA system (R&D Systems, Minneapolis, MN, USA) (21). The inter-assay coefficient of variation was 8.8%. No biological degradation has been described using stored specimens, indicating a high validity for our measurements.

### Statistical analysis

All analyses were stratified by gender to explore heterogeneities in the independent associations of vitamin D with adiponectin. Descriptive analyses stratified by gender and race/ethnicity, age-adjusted correlations, and multivariable linear regression were used to analyze this association. We investigated the effect modification of the BMI categories of lean, overweight, and obese as defined above.

Linear regression models were used to assess the association of vitamin D with adiponectin with adjustment for age, gender, BMI, systolic blood pressure, HDL-cholesterol, triglycerides, FPG, insulin resistance estimated with the HOMA-IR, income, and season of blood drawing.

The dietary intake of vitamin D, available in a subsample of participants (*n* = 167, including 68 AA), was used for correlations with serum vitamin D.

All computations were performed using the SAS software version 9.2 (SAS^®^ Institute, Inc., Cary, NC, USA).

## Results

Among our 426 study participants, the mean (SD) age of the study sample was 50.5 (9) years (Table 1). The mean (SD) levels of vitamin D were 27.4 (9.8) ng/mL in white women, 25.5 (9.3) ng/mL in white men, 16.9 (7.3) ng/mL in AA (AA) women, and 18.8 (7.3) ng/mL in AA men (Table 1). Thus, the average vitamin D levels among AA were in the deficiency category. The mean (SD) levels of adiponectin were 17.0 (17.1) μg/mL in white women, 9.9 (11.3) μg/mL in white men, 6.6 (4.8) μg/mL in AA women, and 9.4 (11.6) μg/mL in AA men (Table 1). Therefore, the AA women had extremely low-average adiponectin levels, due probably to high-BMI levels. AA participants had a higher BMI average, but a lower mean for triglycerides in both genders (Table 1). HDL-cholesterol was higher in AA men, but lower in AA women (Table 1).

**Table 1:** **Sex- and race/ethnicity-specific descriptive characteristics of the study covariates**.

Variable	Whites				African-Americans			
	Men (*N* = 82)		Women (*N* = 136)		Men (*N* = 71)		Women (*N* = 137)	
	Mean	SD	Mean	SD	Mean	SD	Mean	SD
Vitamin D	25.5	9.3	27.4	9.8	18.8	7.3	16.9	7.3
Adiponectin	9.9	11.3	17.0	17.1	9.4	11.6	6.6	4.8
Age	52.6	8.6	51.9	8.4	49.0	8.5	49.0	9.3
BMI	29.9	6.1	27.4	6.5	30.1	6.9	31.8	7.4
SBP	121.5	14.9	117.6	17.6	123.0	18.5	124.4	19.2
DBP	80.0	10.7	74.9	11.0	79.9	11.8	80.3	12.0
HDL-C	47.9	15.2	65.8	17.8	52.4	13.1	61.9	15.3
TG	168.1	112.6	121.6	66.2	99.5	46.3	93.6	40.1
FPG	98.7	39.5	86.7	8.8	90.5	9.5	89.4	18.6
HOMA-IR	3.37	4.02	2.35	2.83	2.55	2.06	3.03	2.57

*SD, standard deviation; BMI, body mass index; HDL-C, high-density lipoprotein cholesterol; TG, triglycerides; SBP, systolic blood pressure; DBP, diastolic blood pressure; FPG, fasting plasma glucose; HOMA-IR, insulin resistance by homeostasis assessment model HOMA-IR*.

*Vitamin D was measured in ng/mL, adiponectin in μg/mL, BMI in kg/m^2^, SBP and DBP in mm of Hg, HDL-C, TG, and FPG in mg/dL*.

The age-adjusted correlations between vitamin D and adiponectin were *r* = 0.38 (*p* < 0.0001) in white women but not significant among white men and among AA. Among whites, vitamin D was statistical significantly correlated with BMI (*r* = −0.28) in both genders, with HDL-cholesterol (*r* = 0.29) in women. Among AA, vitamin D was significantly correlated in women with HDL-cholesterol (*r* = 0.20) and HOMA-IR (*r* = −0.23). Among white men participants, adiponectin was age-adjusted correlated with HDL-cholesterol (*r* = 0.34) and triglycerides (*r* = −0.25), whereas in white women participants adiponectin was correlated with BMI (*r* = −0.36), HDL-cholesterol (*r* = 0.48), triglycerides (*r* = −0.26), and HOMA-IR (*r* = −0.19). Among AA men participants, adiponectin was significantly correlated with HDL-cholesterol (*r* = 0.32) and HOMA-IR (*r* = −0.30), whereas in AA women, adiponectin was correlated with HDL-cholesterol (*r* = 0.41), triglycerides (*r* = −0.25), and HOMA-IR (*r* = −0.28).

There was a borderline effect modification of the association vitamin D – adiponectin by BMI among AA participants by gender subgroups (*p* = 0.05), but not among white participants. Among lean white women (*n* = 63), there was a significant direct association between vitamin D and adiponectin (β = 0.02, *p* = 0.04) after adjustment for age, systolic blood pressure, HDL-cholesterol, triglycerides, income, and season of blood drawing (Table 2). On the contrary, in lean AA women (*n* = 23), there was a significant inverse association (β = −0.06, *p* = 0.01) after the same adjustment (Table 2). These estimates remained unchanged (identical) when additionally adjusting for insulin resistance (HOMA-IR).

**Table 2:** **Associations^a^ between vitamin D and adiponectin stratified by obesity status**.

	Lean participants^b^			Overweight participants^b^			Obese participants^b^		
	β	SE	*p*-value	β	SE	*p*-value	β	SE	*p*-value
**Whites**									
Women	**0.02**	**0.01**	**0.04**	0.01	0.02	0.46	0.04	0.02	0.05
Men	−0.06	0.06	0.43	−0.02	0.01	0.21	0.003	0.03	0.92
**African-Americans**									
Women	−**0.06**	**0.02**	**0.01**	−0.01	0.02	0.50	0.01	0.01	0.20
Men	0.006	0.04	0.89	−0.06	0.05	0.30	0.02	0.03	0.50

*^a^Models were adjusted for age, systolic blood pressure, HDL-cholesterol, triglycerides, income, and season of blood drawing*.

*^b^Lean participants were those with a BMI <25 kg/m^2^, overweight those with BMI ≥25 and <30 kg/m^2^, and obese those with BMI ≥30 kg/m^2^*.

*Bold font indicates main findings of investigation*.

Dietary vitamin D was significantly associated with serum vitamin D in both white women (*r* = 0.30) and white men (*r* = 0.52), but among AA only in women (*r* = 0.43).

## Discussion

### Principal findings

The association of vitamin D and adiponectin is dependent on race, gender, and BMI category. Among lean white women, there was a significant direct association, whereas in lean AA women the association was inverse. No association was present among obese individuals.

### In the context of previous literature

Our results are consistent with the observation that AA have lower plasma levels of vitamin D when compared with their white counterparts. As a recent study indicated, AA in the Southeastern U.S. are four times as likely to have a sub-optimal vitamin D plasma concentration (<15 ng/mL) than their white counterparts (22). A direct association between plasma vitamin D and circulating adiponectin has been suggested by a series of previous studies (15, 16, 23, 24). Some studies showed that the association was increased among participants with higher BMI (15, 24). Nevertheless, other studies failed to identify the same trend, probably due to relatively lean participants in some of the previous studies (16). Our metro Atlanta participants had a relatively high BMI and therefore are well suited to contrast the association vitamin D – adiponectin among those relatively lean and those obese.

Contrary to some previous studies, we did not find a direct association between vitamin D and adiponectin among those participants that are obese. In a recent study with Korean participants (25), after adjusting for age, sex, BMI, smoking, and alcohol intake, serum vitamin D levels showed a significant correlation with adiponectin (*p* < 0.05) level among overweight and obese people (BMI >23) who are likely to be at a risk for cardiovascular disease. In a clinical trial in which 19 vitamin D-deficient peritoneal dialysis patients were treated with cholecalciferol, despite a mean 25(OH)D significantly increase, no change in serum adiponectin after vitamin D replacement was detected (26). Similarly, serum levels of adiponectin were not related with vitamin D and calcium levels in patients with primary hyperparathyroidism (27). Similarly, to our findings in white lean participants, controlling for age, sex, race, sexual maturation, season, physical activity, and percent body fat, 25(OH)D concentrations were significantly correlated with adiponectin (*r* = 0.06, *p* = 0.05) among healthy black and white adolescents residing at southern U.S. latitudes with a year-round sunny climate (28).

### Potential mechanisms

Vitamin D negatively regulates the expression of renin, and thus, the activity of the renin–angiotensin system (29, 30). It appears that adipocytes produce all the components of a local adipose-tissue renin–angiotensin system whose activity in turn inhibits adiponectin secretion (31, 32). Since the activity of the adipose-tissue renin–angiotensin system increases with higher adiposity (33), it was previously hypothesized that increased adipose-tissue renin–angiotensin system activity may represent a potential mechanism for the relative low-adiponectin levels seen in obesity (23). The direct association between vitamin D and adiponectin may be mediated by the negative regulation of the adipose-tissue renin–angiotensin system by vitamin D metabolites. As our results showed a direct association vitamin D – adiponectin only among lean white participants, other mechanisms appear to play a more important role. Among those is the observation that vitamin D insufficiency is related to glucose intolerance and type 2 diabetes (34, 35). It has been shown previously that vitamin D supplementation improves insulin resistance as estimated by HOMA-IR, the homeostasis model assessment for insulin resistance (12). As adiponectin is also generally inversely associated with HOMA-IR, the inverse association between vitamin D and adiponectin observed among our AA participants that were lean could not be attributed to such mechanism. Moreover, as mentioned there was no significant correlation between vitamin D and HOMA-IR, and the magnitude of the association vitamin D – adiponectin estimates among our participants did not change after adjustment for HOMA-IR. Therefore, other characteristics of our sample might play an important role. Among those are the relatively low levels of vitamin D among our AA participants that can be explained by their relatively high-BMI average. One of the clinical characteristics associated with vitamin D deficiency is obesity (36, 37). The association between obesity and vitamin D deficiency is either indirect, as obese individuals tend to have less outdoor activity and thus less sunlight exposure, or direct, as vitamin D is fat soluble and may be sequestered and stored in fat tissues (38), which decreases the bioavailability of vitamin D in the circulation.

Therefore, despite a relatively high-average BMI, among our non-lean women participants and among men participants, no direct association between vitamin D and adiponectin was detected. The lower vitamin D due to higher BMI may also explain the higher adiponectin level. If the adipose tissue is absorbing/metabolizing the vitamin D, then this may lead to adiponectin synthesis and release. Thus, the inverse relationship in lean AA women might be a compensatory response to the “normal,” physiological vitamin D, and adiponectin interaction/interrelationship. Failure to detect this in Caucasians may be a consequence of lower vitamin D stored within the adipose tissue.

### Strengths and limitations

The main strength of our investigation is that this study is among the first on the topic with inclusion of both white and AA participants that have a wide range of BMI values. On the other hand, our study sample is localized to one geographical area, so generalizability inherently cannot be inferred. Another important limitation is the fact that we have a limited sample size. To this should be added the inherent fact that the first stage of the META-Health Study (as well as the clinic visit) has a number of epidemiological limitations (e.g., sampling challenges, non-response bias, social response bias, other general selection bias, etc.) that are not fully accounted for.

## Conclusion

While our study adds to the evidence base regarding the relationship between vitamin D and adiponectin levels in non-white populations, the results raised a number of unintended questions about how vitamin D and adiponectin interact in circumstances when they are not related to weight status. Further research is clearly needed to evaluate the effects of vitamin D therapy on circulating adiponectin in relationship with normal weight and obesity. The design of the present study, unfortunately, lacks the scope to inform if and how randomized clinical trials (RCT) on vitamin D supplementation should be conducted. RCT investigators should weigh in on these and other emerging data that describe the relationships among vitamin D, adiponectin, and individual characteristics such as race, gender, and BMI category.

The inverse association between vitamin D and adiponectin among lean AA women might explain the lower levels of adiponectin among AA due to a possible reduced negative regulation (e.g., of the adipose-tissue renin–angiotensin system) by vitamin D metabolites.

## Conflict of Interest Statement

The authors declare that the research was conducted in the absence of any commercial or financial relationships that could be construed as a potential conflict of interest.
